# Testing New Peptides From *Toxoplasma gondii* SAG1, GRA6, and GRA7 for Serotyping: Better Definition Using GRA6 in Mother/Newborns Pairs With Risk of Congenital Transmission in Mexico

**DOI:** 10.3389/fcimb.2019.00368

**Published:** 2019-10-23

**Authors:** Lizbeth Xicoténcatl-García, Sergio Enriquez-Flores, Dolores Correa

**Affiliations:** ^1^Laboratorio de Inmunología Experimental, Instituto Nacional de Pediatría, Secretaría de Salud, Mexico City, Mexico; ^2^Laboratorio de Errores Innatos del Metabolismo y Tamiz, Instituto Nacional de Pediatría, Secretaría de Salud, Mexico City, Mexico

**Keywords:** clinical aspects, GRA6, GRA7, 3D modeling, perinatal toxoplasmosis, SAG1, serotyping, *Toxoplasma gondii*

## Abstract

*Toxoplasma gondii* variant influences clinical profile in human congenital and ocular toxoplasmosis. Parasite genotyping represents a challenge due to insufficient amount of genetic material of the protozoan in the host samples, and isolates are hard to obtain, especially from pediatric patients. An alternative is serotyping, which is based on the presence of specific antibodies against polymorphic proteins related to virulence; the more widely used are GRA6 and GRA7, but most works report cross reactions among the classical strains (I, II, and III). We designed new peptides of GRA6, GRA7, and SAG1 proteins, with more SNPs among the three clonal strains than those previously designed. This was done by identifying BcR and polymorphic epitopes by means of bioinformatics; then we designed peptides with linkers joining the specific regions and predicted their 3D structure. With the commercial molecules synthesized on the basis of these designs, we tested 86 serum samples from 42 mother/newborn pairs and two congenitally infected newborns, by indirect ELISA. We implemented a strategy to determine the serotype based on scatter plots and a mathematical formula, using ratios among reactivity indexes to peptides. We found low frequency of samples reactive to GRA7 and SAG1, and cross reactions between GRA6 serotypes I and III; we modified these later peptides and largely improved distinction among the three clonal strains. The chronicity of the infection negatively affected the reactivity index against the peptides. Serotyping both members of the mother/child pair improves the test, i.e., among 26% of them only one member was positive. Serotype I was the most frequent (38%), which was congruent with previous genotyping results in animals and humans of the same area. This serotype was significantly more frequent among mothers who transmitted the infection to their offspring than among those who did not (53 vs. 8%, *p* = 0.04) and related to disease dissemination in congenitally infected children, although non-significantly. In conclusion, serotyping using the improved GRA6 peptide triad is useful to serotype *T. gondii* in humans and could be implemented for clinical management and epidemiological studies, to provide information on the parasite type in specific areas.

## Introduction

Toxoplasmosis is the most common parasitic infection worldwide, because it affects all homoeothermic species, including humans (Montoya and Liesenfeld, 2004). It has a strong impact on immunosuppressed individuals, as well as on pregnant women and their babies, because it may cause spontaneous abortions, newborn prematurity and low weight, hepatosplenomegaly, hydrocephalus, intracranial calcifications, psychomotor retardation, hearing loss, and retinochoroiditis (Ambroise-Thomas and Petersen, 2000; Wallon et al., 2014).

*Toxoplasma gondii* was considered a clonal population formed by three classical types (I, II, and III) in Europe and North America, but non-archetypal or “atypical” variants were found in other geographical areas such as South America; actually, close to 300 genotypes have been reported, which are classified in 16 haplogroups distributed within six clades (Su et al., 2012).

Actual evidence is controversial regarding the role of parasite type on clinical outcome, although some studies suggest that type I and atypical strains are more aggressive in congenital cases (Morisset et al., 2008; Rico-Torres et al., 2016). Thus, identification of the parasite may have relevance in terms of prognosis and, as a consequence, clinical management; this is of importance, considering that the effective drug combination provokes serious adverse effects (Montazeri et al., 2017).

To type this parasite, isolates and clinical samples from infected hosts are used, but the former are infrequently obtained and there is reduced amount of parasite DNA in the host tissues. For these reasons, Kong et al. (2003) developed a typing method based on antibody binding to polymorphic peptides, designed from proteins related to virulence. This is a quick and easy method that is performed with serum or plasma, which takes advantage of the natural amplification mediated by the immune response. The dense granule proteins GRA6 and GRA7 are the more commonly used. GRA6 has been characterized as a 32 kDa protein that is localized in the tachyzoite dense granules, and in the intravacuolar network of the parasitic vacuole. GRA7 is a 29 kDa protein, with multiple functions, also associated with the intravacuolar network and the parasite membrane complex. Several peptides derived from these proteins have been used for serotyping cases infected with I, II, or III type strains. Nevertheless, most peptides used do not allow discrimination among them, due to the presence of cross-reactions between type I and III or type II and III.

Another interesting candidate is SAG1, a highly antigenic protein widely used for diagnosis of *T. gondii* infection, which was also tested by Kong et al. (2003); however, there were disappointing results, because neither humans nor animals reacted to the peptides chosen. However, the coding gene is widely used to genotype strains together with other nine loci; thus, it deserved our attention (Su et al., 2010).

In this work we designed new SAG1, GRA6, and GRA7 peptides, considering those previously reported and the antigenic and polymorphic regions of the whole proteins. We tested them by indirect ELISA with positive human serum samples taken from mother-newborn pairs. We found promising results with a specific GRA6 peptide triad and a systematic procedure to establish the serotype.

## Materials and Methods

### Biological Material and Basal Methods

In order to validate the designed peptides, we used *T. gondii* positive serum samples from a bank of the Laboratorio de Immunología Experimental of INP, firstly 14 from pregnant women and six from neonates; and then 86, 42 from women/offspring pairs plus two congenitally infected children. They were recruited through pre or postnatal screening projects, or they were clinical cases attending INP for clinical management (Gómez-Toscano et al., 2018). The mothers were 17–39 years old (mean and median = 27 years) and lived in the Plateau of Mexico. The offspring were 30 males and 14 females, with an age range of zero-730 days, and percentiles 50/70 = 13/38 days. Thirty-two congenitally infected babies were studied, twenty with moderate/severe clinical problems, including microcephalia or hydrocephalus, retinochoroiditis, and/or splenomegaly/hepatomegaly; these patients had a bad outcome, defined as a poor response to treatment, with major sequels and in three cases, death. Thirteen children had less severe clinical presentation, including retinochoroiditis, and mild to moderate psychomotor retardation; they responded finely to treatment and neuro-psychomotor stimulation; one case was not classified, because he did not continue in the study. Most of the cases are described in detail elsewhere (Gómez-Toscano et al., 2018), as well as the criteria to define congenital transmission; briefly, all mothers were positive against *T. gondii* by different serological techniques, including indirect ELISA and western blot against crude antigens. They were also tested for IgG avidity, titration of sequential samples, and endpoint/real-time PCR. Congenital infection was confirmed by IgM, IgG neo-antibodies, titer increase in serial samples, or by end point/real time PCR. The detailed genotyping results of the cases included herein to compare with serotyping, were reported previously (Rico-Torres et al., 2018). The results are based on the PCR-RFLP method of markers *SAG1, SAG3, BTUB, GRA6, c22-8, c29-2, L358, PK1*, and *Apico* reported by Su et al. (2010) followed, when possible, by sequencing. We performed the laboratory procedures as described before (Pujol-Riqué et al., 1999; Kompalic-Cristo et al., 2007; Cañedo-Solares et al., 2009; Caballero-Ortega et al., 2014; Gómez-Toscano et al., 2018).

### Bioinformatics for Peptides Design and 3D Modeling

The *SAG1, GRA6*, and *GRA7* gene sequences (Supplementary Table 1) were first identified at DNA level using multiple sequence alignments in the MUSCLE server (Edgar, 2004). Then, the protein sequences were searched, and the translated polymorphisms marked. To eliminate the signal peptide, as well as the intra-membrane and hydrophobic regions we used servers SignalP 4.1 (Petersen et al., 2011), TMHMM 2.0 (http://www.cbs.dtu.dk/services/TMHMM-2.0/) and Protscale (Gasteiger et al., 2005), respectively. Finally, the hydrophilic and B-cell epitope segments were determined with ABCpred server using a score ≥ 0.8 as cutoff (Saha et al., 2005).

**Figure 1:**
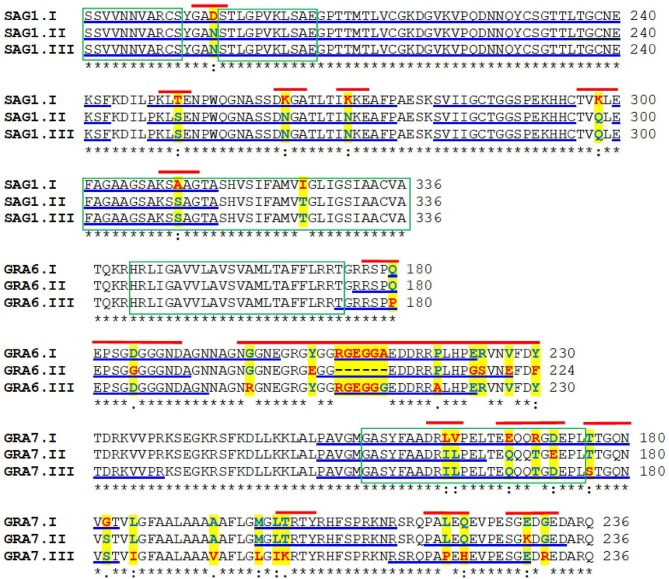
Multiple alignments of the amino acid sequences of the protein variants of *Toxoplasma gondii* SAG1, GRA7, and GRA6. The amino acids highlighted in yellow indicate the polymorphisms; those underlined in blue mark potential B cell epitopes. The green boxes indicate hydrophobic regions and the red lines show the short segments used to design the serotyping peptides.

On the basis of the criteria mentioned above, we designed synthetic peptides with non-contiguous segments joined through a glycine/serine 10 amino acid-long linker [obtained from a sequence reported by He and Taussig (1997)], to prevent steric impediment among fragments of interest. Due to the results with the three original GRA6 peptides with the first 20 samples (see below), we designed new type I and III peptides from this protein, eliminating a conserved region between them; so, we ended up using five GRA6 peptides: one for type II and two of each type I and III molecules, with (Wi) and without (Wo) the conserved region (see Figure 1). A cysteine residue was added to all peptides at the amino terminus, in order to bind them to the special ELISA plates mostly in a vertical way.

The secondary structures of the peptides designed were predicted with the Advanced Protein Secondary Structure Prediction Server (Raghava, 2002). To obtain the three-dimensional models, we used the ITASSER web server (Zhang, 2008), which is based on multiple alignments of homologous sequences with the target. After obtaining the resulting models, each one was validated with the Protein Structure Validation server (Huang et al., 2005); those more congruent with the secondary structures predicted before, were chosen for modeling the 3D structure with the molecular graphics PyMOL (DeLano, 2002).

### Peptides and ELISA

A total of 10 peptides containing the chosen sequences of SAG1, GRA6, and GRA7 (Figure 1) were commercially synthesized by Invitrogen (Texas, USA) or Biosynthesis (Texas, USA) with 95–99% purity, as demonstrated by mass spectroscopy. The lyophilized peptides were stored at −80°C until use.

For ELISA, Thermo Scientific Immobilizer Amino plates (Nunc Co, Denmark) were sensitized with 100 μL/well of the peptides at 10 μg/mL of 15 mM sodium carbonate buffer, pH 9.6 and incubated at 4°C overnight. Among steps, wells were washed five times with 200 μL/well of 10 mM phosphate buffered, 0.15 M saline, pH 7.2, plus 0.05% Tween 20 (PBS-T). Non-specific binding sites were blocked with 200 μL/well of 0.3% bovine serum albumin in PBS-T for 1 h at 37°C. The serum samples and the goat anti-human IgG-peroxidase conjugate (Sigma-Aldrich Corp., St Louis, MO, USA) were diluted 1:1,000 in PBS-T, and incubated for 2 h at 37°C. A solution of Ortho-phenylendiamine (5 mg) /30% H_2_O_2_ (4 μL) in 10 mL of 0.1 M sodium citrate/citric acid buffer, was used to develop the reaction, which was stopped by addition of 50 μL/well of 0.1 N sulfuric acid. Absorbance values were obtained in an ELISA reader (Turner Biosystems 9300-010, Sunnyvale, California) at 490 nm and captured with the Modulus TM Microplate Reader program.

### Analysis and Statistics

The reactivity index (RI) of each sample against every peptide, was calculated by dividing the mean absorbance of duplicates of each sample, by the average plus three standard deviations of ten *T. gondii* crude antigen-negative controls, tested in the same plate; an RI≥1.0 was considered positive. The serotype was pre-defined using scatter plots which compared the RI obtained for a given peptide vs. the RI to a different peptide, for each serum. The Spearman test was used to determine the correlation among RIs found with the three types of peptides. Also, a ratio was built up by dividing the RI for a given peptide-type by the RI for each of the others. A ratio ≥1.15 was considered specific for the peptide in the numerator; e.g.,: RI type to I/RI type to II = 1.26, plus RI to type III / RI to type II = 1.10, and RI to type I/RI to type III = 1.21; the resulting serotype is I.

## Results

Figure 1 shows the complete amino acid sequence of *T. gondii* SAG1, GRA7, and GRA6 in which the regions of interest are marked. As it can be seen, the main antigenic and polymorphic regions are in the second half of the proteins.

**Figure 2:**
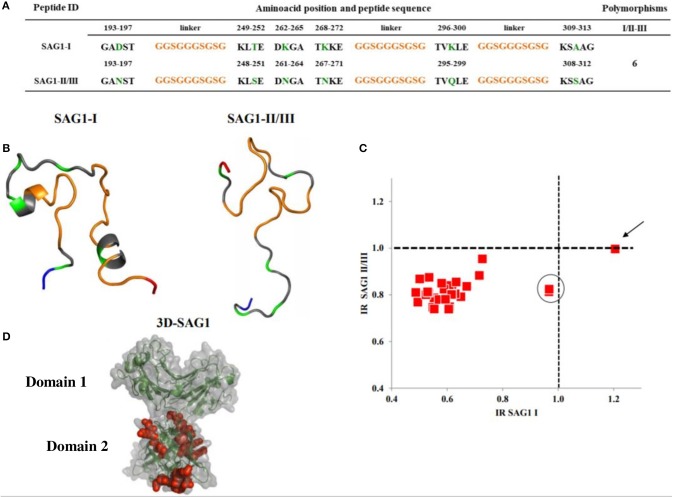
Sequences, 3D models and reactivity of SAG1 peptides with human *T. gondii* positive serum samples. In **(A,B)**, the orange segments represent the linkers; the green ones are the polymorphic amino acids between types I and II/III. The rest, in gray, represent the conserved regions. The NH+ and COO- termini in the 3D models are depicted in blue and red, respectively. A cysteine residue at the NH+ end was added for ELISA plate binding (not shown). **(C)** Reactivity scatter plot of samples with type I and type II/III peptides. The cut offs are marked with vertical and horizontal dotted lines. One sample typed as I, is marked with an arrow. Two other samples potentially also type I, are within the circle. **(D)** Crystallographic structure corresponding to SAG1 type I entire molecule, in which the peptides used were bioinformatically located and are marked in red.

**Figure 3:**
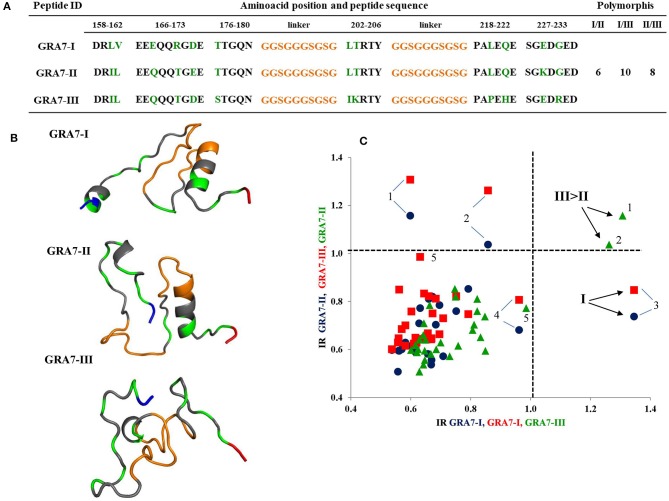
Sequences, 3D models and reactivity of GRA7 peptides with human *T. gondii* positive serum samples. In **(A,B)** the orange segments represent the linkers and the green residues are the polymorphic amino acids among types I, II, and III; gray zones represent the conserved regions. The NH+ and COO- termini are depicted in blue and red, respectively. A cysteine residue at the NH+ end was added for ELISA plate binding (not shown). **(C)** Reactivity scatter plot of samples with type I, II, and III peptides. The cut offs are marked with vertical and horizontal dotted lines. Three positive and two “gray zone” samples (4 and 5) are labeled with Arabic numbers.

**Figure 4:**
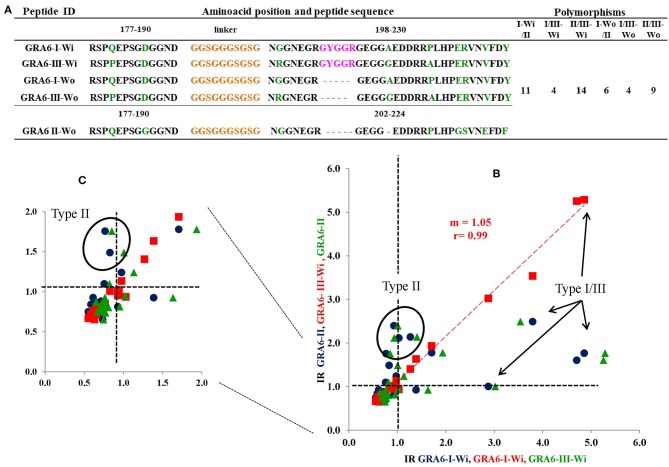
Sequences of all GRA6 peptides and reactivity of human *T. gondii* positive serum samples with three peptides (those I and III presenting the conserved sequence, Wi). In **(A)** the orange parts represent the linkers; the green ones represent the polymorphic amino acids. The pink region is shared by type I and type III original molecules (Wi) and is absent in the type II and types I and III “Wo” peptides. The rest of sequences in gray represent conserved regions among the three molecules. The NH+ and COO- termini are in blue and red, respectively. The cut offs are marked with vertical and horizontal dotted lines. **(C)** is an insert of the results in **(B)**. Examples of cases typed as I/III are marked with arrows, while those typed as II are encircled. Spearman's test was significant for correlation between Type I -Wi and Type III-Wi peptides (*P* < 0.001); *n* = 20.

**Figure 5:**
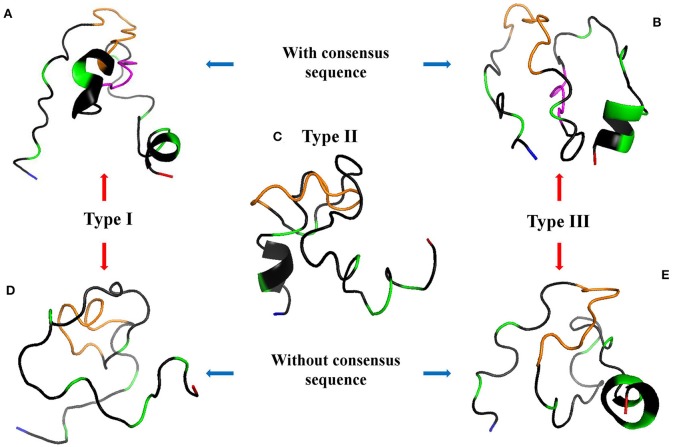
3D models of GRA6 peptides with **(A,B)** and without **(D,E)** conserved region; type II peptide naturally lacks this region so it is at the center **(C)**. The conserved sequence between variants I-Wi and III-Wi (in pink, **A,B**) was eliminated in the Wo forms **(D,E)**. NH+ and COO- termini are depicted in blue and red, respectively; the orange parts represent the linkers; the green ones are polymorphic amino acids and the gray zones represent conserved regions among the five peptides.

The sequences of the peptides designed, their 3D models and the scatter plots of the serological results are shown in Figures 2–5. Six polymorphisms at the protein level were present between the type I and the II/III SAG1 peptides (Figure 2A); the predicted 3D structure of the type-I peptide presents two alpha helixes, one close to the amino terminus, which covers the last section of the linker sequence and residues 249–252 (Figure 2B), and one in the fifth fragment at positions 296–300, closer to the carboxyl end. The prediction of the SAG1-II/III model yielded a long loop. Only one sample gave clear positive RI when tested with these peptides, and it was defined as type I (Figure 2C, arrow); two other samples could be reacting to this same peptide, since their RI is close to but below the cutoff (circle). So, most cases were negative to SAG1 peptides. To understand this better, we predicted the position of the peptides within the known 3D structure of the entire protein, based on crystallographic studies (using PyMOL Protein Data Bank 1YNT; Graille et al., 2005); as it can be seen in Figure 2D, the segments fit below the exposed face of the protein and close to the parasite membrane.

Three peptides of GRA7 were synthesized, which were within a region from position 158 to 233 (Figure 3A). According to the sequence, there are less polymorphisms between type I and type II peptides, since they differ in 6 residues only, and both present an α-helix which starts in the second linker, while peptide type III has ten and eight polymorphic residues when compared to the former two; its 3D structure is also unique, since it does not present complex secondary structures (Figure 3B). Despite these differences, two of the three cases who gave seropositive results, cross-reacted between type II and III peptides, and the third was clearly identified as serotype I (Figure 3C). Besides, there were two other samples (marked as 4 and 5 in Figure 3C) that reacted close to the cutoff, which could be typed as I and III, respectively, but as in the case of SAG1, most samples were non-reactive.

Figure 4A depicts the amino acid sequence of the five GRA6 peptides designed. As it can be seen, types I and III differed in four residues (either with or without the conserved -pink- region). GRA6-II does not have that conserved region and its sequence differs in eleven and fourteen residues with GRA6-I-Wi and III-Wi peptides, respectively. Using these peptides, a clear distinction was observed between sera reacting to type II and types I or III, but a strong cross-reaction was present between the latter two (red points in Figure 4B).

The 3D model of GRA6-I-Wi presents two alpha helixes (Figure 5A); the first includes the last glycine of the linker and five residues from position 198 to 202 (GNGGNE) while the second starts at position 226 in the COO- region (NVFD). The model of GRA6-II has an incomplete alpha-helix close to the NH+ end (aas 179-184: PQEPSG) absent in all other peptides (Figure 5C); GRA6-III-Wi has an alpha helix in the 223–229 segment (ERVNVFD) larger than that of GRA6-I-Wi.

Since type I-Wi and III-Wi presented strong cross-reaction partially explained by the five-amino acid conserved sequence (pink regions), we decided to synthesize and test both peptides without this region, in order to enhance their differences (Figures 5D,E). As a result, there were changes in the predicted 3D structure of peptide type I, which lost an alpha-helix at the carboxyl terminus, while no apparent structural change was observed in the case of peptide III, besides the shorter length (Figure 5E). This modification decreased variations only in five amino acids in relation to peptide type II (Figure 4A).

**Figure 6:**
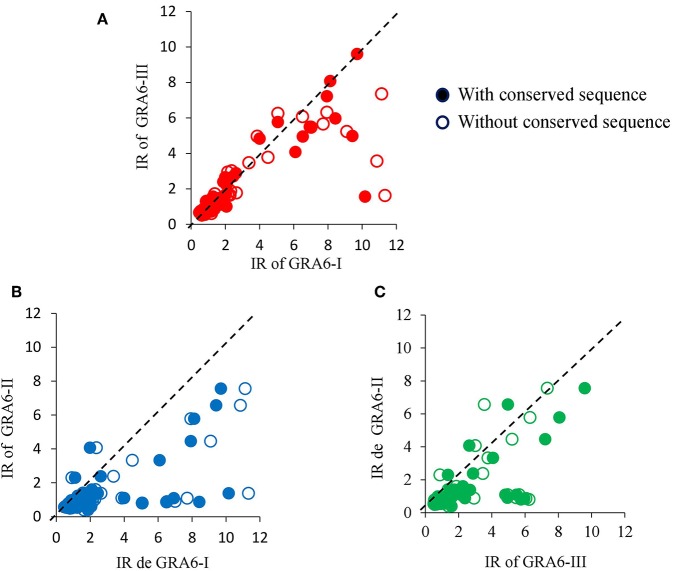
Effect of elimination of conserved sequence of type I **(A,B)** or type III **(A,C)** peptides on reactivity of serum samples from mother/newborn pairs positive for *T. gondii* crude antigen. The dotted line within each graph is the theoretical perfect correlation; *n* = 86, 42 mother/children pairs plus two newborns.

**Figure 7:**
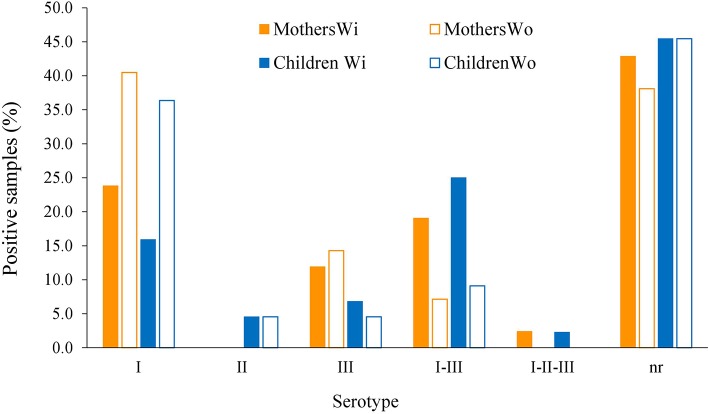
Global effect of GRA6 I and III conserved sequence elimination on frequency of the serotypes. Samples from mothers (orange bars, *n* = 42) and offspring (blue bars, *n* = 44) were tested against the peptides with (closed bars) or without (open bars) the conserved sequence shared by the classical strains type I and III and absent in type II strains (see pink regions in Figures 4, 5, upper panels).

Generally, an increase in the RI to type I peptide and a decrease to type III molecule was observed when using the GRA6-Wo peptides for serotyping (Figures 6A,B); this resulted in an augmented frequency of serotype I positive samples, and a decrease in type III, double positive (“I–III”) and non-reactive samples (Figure 7). Conversely, a lower discrimination between type III-Wo and type II peptides was observed for some samples (Figure 6C). Globally, better definition of serotype was obtained in 25.6% of cases by removing the conserved sequence in type I and III peptides (Table 1).

**Table 1:** Changes in serotype due to elimination of the conserved sequence of GRA6 peptides I and III.

**Effect**	**Wi**	**Wo**	***n***	**Percent (%)**
No change	I	I	14	68.6
	II	II	2	
	III	III	6	
	I–III	I–III	2	
	nr	nr	35	
Better definition	I–III	I	16	25.6
		III	1	
	I–II–III	I	1	
		I–III	1	
	nr	I	1	
		I–III	2	
Lower definition or change	I	nr	1	5.8
		III	1	
		I–III	1	
	III	I–III	1	
		I	1	

**Figure 8:**
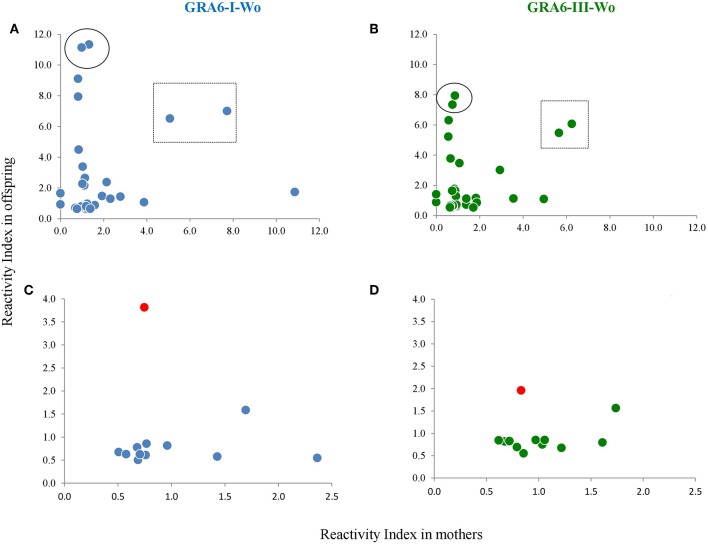
Scatter plots of mother vs. offspring RIs against type I and type III peptides without the conserved sequence in the group of congenital infection **(A,B)** and that of mothers who did not transmit the infection to their offspring **(C,D)**. One pair is marked in **(C,D)** as a red dot, because the baby presented higher RI values than the mother. The pairs enclosed in a square or a circle in **(A,B)** are examples of pairs which samples were taken simultaneously from the mother and the child. The regression line equation as well as the Spearman coefficients are shown for the lower panels, discarding the outlier case. No correlation was observed between mothers and offspring in the group of vertical transmission (not shown); *n* = 42 pairs.

We compared the serotyping results between mothers and their offspring; representative examples of RI results using type I and type-III Wo peptides are shown in Figures 8A,B. As it can be seen, there is no correlation in the congenital transmission group; i.e., except for two pairs (within a square), only the mother or only the baby had high antibody levels to any given peptide. It should be noted that these samples were taken from the mother and the children at the same time, but this was also true for other cases (like those encircled). In cases with no congenital transmission, the maternal RI was always higher than that of her child, with the exception of pair 10, because the sample of the baby had higher values than those of the mother (marked in Figures 8C,D in red). These results were also observed with other GRA6 peptides (not shown). The sample of the mother was taken 30 days after that of the baby.

The use of both mother and child serum samples together with the Wo peptides led to higher proportion of parasite variant identification, i.e., the serotype matched between the members in 6 and 12 pairs, with the Wi and Wo peptides, respectively; also, 21 and 18 variants could be identified in the mother or the baby with the Wi and Wo peptides (Table 2). Moreover, there was a decrease in partial matches from 11.9 to 4.8%, and a global decrease in mother/child mismatches from 50.0 (31.0 + 19.0%) to 42.9 (28.6 + 14.3%). Only ten pairs were non-reactive to all peptides; this was not related to vertical transmission, dissemination of congenital infection, or time difference between maternal and offspring sampling (not shown).

**Table 2:** Match of serotype in mother-offspring pairs using the peptides with and without the conserved sequence.

**Match**	**Serotype in**		**With cons. seq**.			**Without cons. seq**.		
	**Mother**	**Child**	***n***		**Percent** **(%)**	***n***		**Percent** **(%)**
Perfect	I	I	2	16	38.1	10	22	52.4
	III	III	1			0		
	I–III	I–III	3			2		
	nr	nr	10			10		
Partial	I	I–III	3	5	11.9	0	2	4.8
	III	I–III	0			2		
	I–III	I	2			0		
Missmatch	I	III	2	13	31.0	2	12	28.6
	III	II	1			5		
	I	nr	3			0		
	II	nr	0			1		
	III	nr	3			3		
	I–III	nr	3			1		
	I–II–III	nr	1			0		
	nr	I	2	8	19.0	6	6	14.3
	nr	I–III	5			0		
	nr	I–II–III	1			0		
Total			42			42		

**Figure 9:**
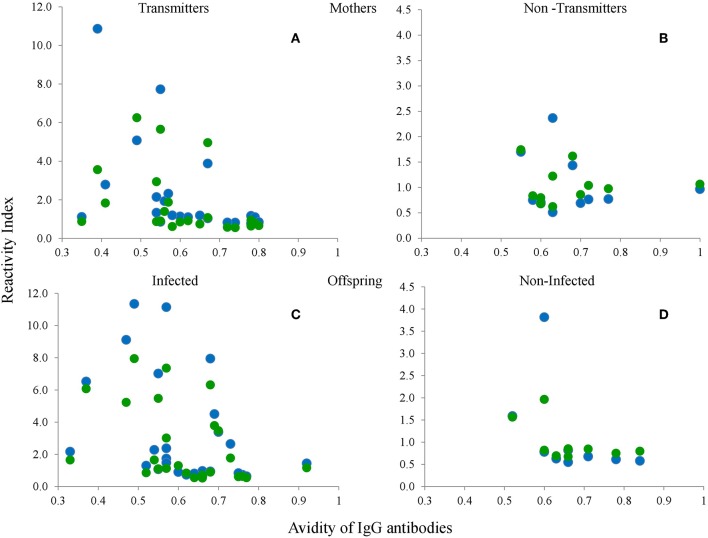
Relation between avidity of IgG antibodies against the crude extract and RIs of serum samples from the mothers **(A,B)** and the babies **(C,D)**, of the groups with **(A,C)** or without **(B,D)** vertical transmission (congenital infection) against the type I-Wo (blue circles) and type III-Wo (green circles) peptides; *n* = 42 pairs.

In order to get an insight as to whether the reactivity to the peptides was related to the time of infection, we determined its relation to antibody avidity, and found there was a tendency for a negative relation, i.e., a lower response to the peptides was observed in chronic samples; nevertheless this relation was not statistically significant (Figure 9).

**Figure 10:**
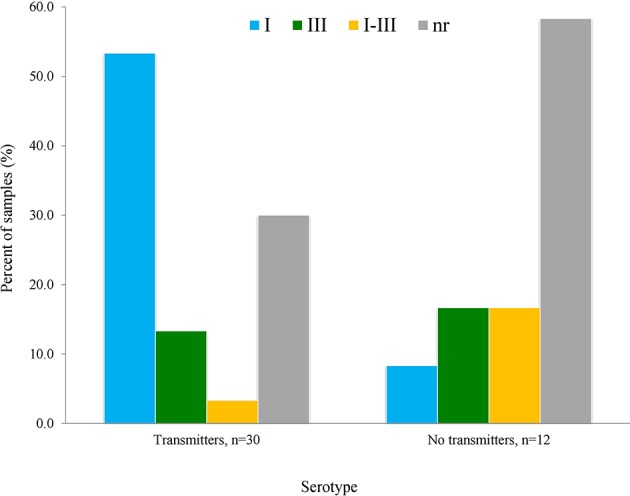
Relation of vertical transmission of *T. gondii* with serotype as determined using the serum samples from mothers, using the formula explained in Materials and Methods; *n* = 42 pairs. The difference of patters between groups was statistically significant (*P* = 0.04).

**Figure 11:**
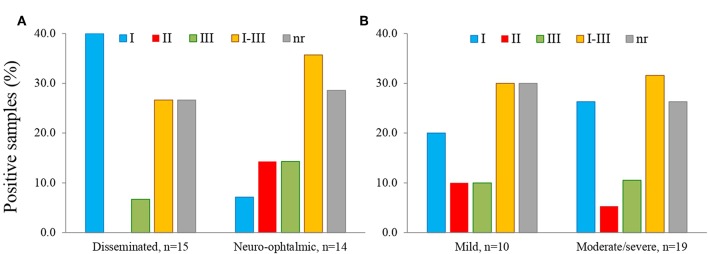
Relation of serotype with location **(A)** and severity **(B)** of congenital toxoplasmosis in the infected offspring; *n* = 29.

We observed that serotype I was significantly related to vertical transmission (Figure 10; *P* = 0.04). The same variant was more frequent among congenitally infected children with disseminated disease than among those cases with localized lesions (neuro-ophthalmic); moreover, type II (non-virulent) type was absent in the former group (Figure 11A). The severity of the disease did not seem related to a particular serotype (Figure 11B).

Table 3 shows the serotype of the nine mother/offspring pairs that we could genotype (reported previously in Rico-Torres et al., 2018). The aim of this table is to depict the concordance between genotype and serotype and the better definition of a serotype by using either GRA6-Wi or GRA6-Wo in mother and child samples. In three pairs we found a perfect concordance between *GRA6* genotype and GRA6 serotype in either the mother or the child, including one case with a double infection was demonstrated by the presence of serotype I in the mother and genotypes I and II in the child (pair 39). In pair 41, a double infection can be observed; the child had global genotype I and III but only genotype I was detected for the *GRA6* locus; we demonstrated the presence of serotype III with the sample of the mother. In other four cases, a partial concordance between the global genotype and serotype was observed; e.g., pair 36 both mother and child had GRA6 serotype I, but the mother's global genotype was I+II which was also a double infection.

**Table 3:** Geno and serotype in nine mother newborn pairs with congenital toxoplasmosis.

**Pair**	**Person**	**Genotype**	***GRA6* genotype**	**Serotype GRA6 Wi**	**Serotype GRA6 Wo**
2	Mother	**I, II**	**I**	**I**	**I**
	Child			I-III	**I**
16	Mother	I	I	nr	nr
	Child			nr	nr
17	Mother			I-III	**I**
	Child	**I**		**I**	**I**
19	Mother	**I, III**		**I**	**I**
	Child			**I**	**I**
23	Mother	**I, II, III**		**I**–**III**	**III**
	Child		**III**	I	**I**–**III**
36	Mother	**I**+II^*^		I–III	**I**
	Child			I–III	**I**
37	Mother	**I** (+II)^*^		**I**	**I**
	Child	II		III	III
39	Mother			**I**	nr
	Child	I+II^*^	I+II^*^	nr	nr
41	Mother			**III**	**III**
	Child	I+III^*^	I	nr	nr

* *These patients harbored double infections. Genotypes are summarized from data published before (Rico-Torres et al., 2018). Those cases with complete or partial concordance are in bold type*.

## Discussion

In this work, we designed new peptides of *T. gondii* proteins GRA6, GRA7, and SAG1 based on the peptides previously described by Kong et al. (2003). Most of the selected regions they used were at the carboxyl terminal region, especially for GRA6 and GRA7, with deficient distinction between the classical lineages I and III or I and II, since they are very similar and, in some regions, identical. They also used SAG1 protein peptides but observed low reactivity. Thus, our purpose was to add polymorphic and antigenic regions of the entire proteins, in order widen the possibility of distinguishing among the three classical lineages I, II, and III.

The SAG1 protein is immunodominant and widely used for serological diagnosis, hence we expected good results; however, the peptides used in this study only reacted with up to three samples (serotype I). Thanks to the availability of crystallized 3D structures and the bioinformatic analysis, we could observe that the peptides used are located in a region that is close to the cell membrane, so they may not be accessible for the B-cell receptor *in vivo*, despite having chosen potential antigenic epitopes for B cells (Grimwood and Smith, 1992; Graille et al., 2005). Besides, some reports show that the sera of infected people recognize more strongly the D1 domain, which is highly conserved (Godard et al., 1994). In summary, SAG1 is an excellent option for diagnosis, but it does not seem useful for serotyping.

The peptides based on GRA7 used in the present study included six more polymorphisms than those previously reported (Kong et al., 2003; Sousa et al., 2009; Xiao et al., 2009). However, they did not turn out to be immunodominant, since most of the samples had no reaction, despite the bioinformatic prediction; in fact, the RI obtained for the few positive samples was low. Serotype I was found in one case (sample 3 in Figure 3C); the amino acids responsible for this reaction could be those of the 158–180 aa region, where the type I peptide has unique polymorphisms. Kong et al. (2003) used a region within 166–173 aa for type I, but they found no positive sample and did not analyze the same region for strains II and III. On the other hand, they found 50% positive samples using the 225–233 aa region which is the carboxyl terminal part of the protein for peptides II and III, but they did not use the corresponding type I peptides; moreover, they found mainly type II reactive samples, although with some cross-reactivity against type III. We observed the opposite: a stronger reaction to type III with a lower RI for peptide II. Anyway, we mostly found negative samples, for which we have no certain explanation. Both the results of Kong et al. (2003) and ours can be derived partially from the strains that prevail in the corresponding geographic areas, that is, North America and Mexico, respectively (Dubey et al., 2004; Cedillo-Peláez et al., 2011; Rico-Torres et al., 2018). The high rate of non-reactive samples in this study could be due to technical reasons, since the cut-off point used by Kong et al. (2003) was less strict compared to ours; however, if we set a less value, only two other positive samples would become positive (samples 4 and 5 in Figure 3C). Unfortunately, other studies reporting successful results used few human samples (Sousa et al., 2009; Xiao et al., 2009). One explanation of low RIs is possible negative influence of the linker region, which even formed part of secondary structures in types I and II (see Figure 3B), probably impeding antibody recognition. Further designs with these molecules deserve attention, since they are derived from a protein related to virulence.

Based on previous works, in which peptides from the carboxyl end of GRA6 (220–230 aa) were used, we decided to add some others located at the center of the protein sequence, adding in this way five polymorphisms. With them, it was possible to discriminate between variants II from I or III; but as in other studies, it was not possible to distinguish between the latter two (Kong et al., 2003; Peyron et al., 2006; Sousa et al., 2008, 2009). Therefore, we focused on a region (205–209 aa) conserved in peptides I and III, and absent in the type II molecule, which could be immunodominant and therefore masking the polymorphisms between them. The desired discrimination was attained in many cases, since a considerable number of I/III sero-variants were defined as type I and, to a lesser extent, type III (see Table 1).

The 3D prediction showed that peptide GRA6-I loses two coils when the conserved sequence is absent, one of them in the COO- term, shared with GRA6-III; this suggests, on one hand, an allosteric effect of the preserved region, and on the other hand, that the loss of these small loops exposes the linear polymorphic amino acids. Conversely, the reactivity against peptide III decreased so that cross reactions with type II molecule appeared, despite the largest number of polymorphic amino acids were between them (see Figures 1, 4, 5). Some authors had suggested that cross-reactivity between peptides of classical strains was due to infections with atypical variants in humans, cats, sheeps and pigs (Nowakowska et al., 2006; Sousa et al., 2009; Maksimov et al., 2012). Our data shows otherwise, i.e., that cross-reactions do exist among classical strains, most probably because of the conserved amino acids.

Some samples reacted with several peptides, despite their modification; although this may be due to unsolved cross-reactions, it may also reflect infections with two or more strains, which we demonstrated in a small group of cases recently genotyped in Mexico City and included in the last table herein. Cross-reactive samples may also reflect infections by atypical strains; we have not found these strains in the region of Mexico from where these cases were recruited, but this cannot be ruled out (Rico-Torres et al., 2018).

Paradoxically, it has been proposed that the absence of reaction to any of the three peptides is due to atypical variants (Maksimov et al., 2012; Shobab et al., 2013). However, our data suggest that this is not the case, since some non-reactive samples were converted to type I or III, and a type I sample became non-reactive using the GRA6-Wo peptide. Also, some genotyped cases shown in Table 3 were serologically non-reactive. The lack of reactivity may be due to several reasons, among them a decay in antibody levels during the chronic phase. In this regard, Maksimov et al. (2018) showed a good response to peptides in recent infections in turkeys and chickens experimentally infected with tachyzoites, which decreases and even becomes negative after 9 weeks. For this reason, we analyzed the correlation between IgG antibody avidity and the RI against each peptide and observed a negative relation; that is, the greater the avidity or chronicity, the lower the RI; thus, our results support that serotyping must be performed during the acute phase. This is reinforced by absence of correlation between mothers and children except for those pairs in which there was no congenital infection. On the other hand, certain individuals (e.g., the mother) may lack receptors for specific epitopes, while others (e.g., the baby) have them, or vice versa. Our results strongly support the use of serial samples and both mother and newborn samples to increase serotyping success.

Interestingly, we found a child diagnosed as not infected congenitally by routine methods of our laboratory, who reacted to the peptide while the mother did it but with low RI (see Figure 8). This suggests that the use of peptides could unmask infections and thus have high diagnostic value for cases with clinical data of congenital infection and with negative routine test results. We have reported something similar when searching IgG antibody subclasses in mother/newborn pairs, which demonstrates that each individual of the twosome reacts independently (Cañedo-Solares et al., 2008). Nevertheless, the sample of the mother was taken 30 days after that of the baby, so antibodies in the later could still be due to maternal transfer during pregnancy.

We observed that serotype I is the most prevalent in the cases studied, which is consistent with previous results in humans, chickens and squirrel monkeys of the same region (Dubey et al., 2004; Cedillo-Peláez et al., 2011; Rico-Torres et al., 2018). A systematic review showed that *T. gondii* type I strains are commonly transmitted before week 24 of gestation, which is a period of greater risk for the development of clinical problems in the baby; in fact, the same study revealed a strong association between type I or atypical variants and clinical problems in the offspring (Rico-Torres et al., 2016). Xiao et al. (2009) suggested a relation between “serotype I” infection in pregnant women (using GRA5, GRA6, and GRA7 derived peptides), and psychosis development in their congenitally offspring decades later. This article supports the notion that the parasite type, especially type I, may be related to clinical problems in congenitally infected individuals. The case series reported recently by us pointed to a more aggressive form of congenital toxoplasmosis in this area of Mexico, since the children are born with symptoms even in those cases detected by pre or postnatal screening and prophylactically treated (Gómez-Toscano et al., 2018). Future serotyping analysis in regions were type II strains are more frequent are needed, since they were underrepresented in this study. Nevertheless, our results support the notion that type I strains were related to vertical transmission and -less clearly- to a broader dissemination in the children.

## Concluding Remarks

Our results support the importance of detailed bioinformatic designs to improve or discard certain molecules for serotyping. GRA7 peptides 3D models revealed structural problems and thus possible ways to improve their performance; also, SAG1 peptides location within the entire protein structure suggests this is not a good candidate for serotyping, despite its immunodominance. Also, the systematization of mathematical and graphical procedures to discriminate among *T. gondii* strains is important: plotting results of RIs vs. each peptide against the other two, may point to the serotype of a given case at first glance, and the use of the formula presented, allows systematization without bias of perception-based decisions. Partial agreement between the genotype and the serotype with the GRA6 “Wo” peptides further supports these strategies. Serotyping efficacy may also be increased considering aspects such as the chronicity of the infection (avidity of antibodies) and the use of additional proteins related to virulence (Sánchez et al., 2014; Maksimov et al., 2018).

From 86 samples and 42 mother/offspring pairs included in this study 51 (59%) and 32 (76%) could be serotyped, respectively, while only eleven cases (13%) and nine pairs (21%) were genotyped. Thus, serotyping is a good support for *T. gondii* variant identification, as suggested by the pioneers of this idea (Kong et al., 2003).

## Data Availability Statement

The results supporting the conclusions of this article are included within the article. The database can be made accessible upon request.

## Ethics Statement

The project that gave rise to this work was carried out according to ethical principles conducted in accordance with the World Medical Association's Declaration of Helsinki. It was approved by the Reviewing Board of the Instituto Nacional de Pediatría of the Ministry of Health of Mexico (INP; IRB-NIH numbers IRB00008064 and IRB00008065), which includes the Investigation, Ethics and Bio-safety Committees (registration number 2012/112). It was based on a serum bank obtained for research purposes, so consents were obtained accordingly.

## Author Contributions

DC wrote the project which gave rise to this article. LX-G and SE-F designed the synthetic peptides. LX-G carried out the serotyping tests. LX-G and DC analyzed the data and wrote the first draft and various versions of the manuscript. All authors reviewed the final version of the manuscript.

### Conflict of Interest

The authors declare that the research was conducted in the absence of any commercial or financial relationships that could be construed as a potential conflict of interest.

## Abbreviations

SAG1: Surface Antigen 1
GRA6: granule dense 6
GRA7: granule dense 7
IVN: intravacuolar
PV: parasitic vacuole
Wi: with the conserved sequence
Wo: without the conserved sequence
RI: reactivity index
nr: non-reactive.
