# Supplementary material for: Testing New Peptides From Toxoplasma gondii SAG1, GRA6, and GRA7 for Serotyping: Better Definition Using GRA6 in Mother/Newborns Pairs With Risk of Congenital Transmission in Mexico
Source: Front Cell Infect Microbiol. 2019 Oct 23;9:368. doi: 10.3389/fcimb.2019.00368 (PMC6819317; doi:10.3389/fcimb.2019.00368)
Supplement: Supplementary file 1 [file Table_1.DOCX]

Supplementary table1. *Toxoplasma gondii* GRA6, GRA7 and SAG1 accession numbers of amino acid sequences

|  |  |  |  |  |  |
| --- | --- | --- | --- | --- | --- |
| Strain | Type | Protein and access number in Genbank | | | |
|  |  | SAG1 | GRA6 |  | GRA7 |
| RH | I | ACT64638.1 | L33814.1 |  | DQ459443.2 |
| ME49 | II | AFO54849.1 | AF239285.1 |  |  |
| BEVERLEY | II |  |  |  | EU157141.1 |
| VEG | III | AFO54871.1 |  |  |  |
| C56 | III |  | AF239284.1 |  | DQ459458.2 |
